# Preamble from the Guest Editor of Special Issue “DFT Quantum-Chemical Calculation of Metal Clusters”

**DOI:** 10.3390/molecules26020442

**Published:** 2021-01-15

**Authors:** Oleg V. Mikhailov

**Affiliations:** Department of Analytical Chemistry, Certification and Quality Management, Kazan National Research Technological University, K. Marx Street 68, 420015 Kazan, Russia; olegmkhlv@gmail.com

As known, the concept of “cluster” is collective and includes substances that are quite diverse in composition and chemical structure. According to the classic definition of this term, the author of which was one of the world’s largest experts in the field of coordination chemistry, Prof. F.A. Cotton, clusters are chemical compounds containing such metal atoms, which are fully or largely covalently bonded to each other, even if the compound contains additional nonmetal atoms that are part of any structural groups associated with metal atoms. Initially, this term referred almost exclusively to the coordination compounds of *p*-, *d*-, and *f*-elements, but over time, more and more new compounds were included in it, although it is worth noting that, in accordance with this definition, under this concept fall primarily those chemical compounds, which include only the atoms of metal elements. This term began to denote, in particular, such compounds as fullerenes and polynuclear compounds formed by boron atoms. Although the term “cluster” has become widely used relatively recently, the very concept of a small group of atoms, ions, or molecules is natural in chemistry, since it is connected with the formation of nuclei during crystallization or associates in a liquid. Owing to this circumstance, later, agglomerations of molecules of the same type were also attributed to clusters–this is how, e.g., “clusters of water molecules” appeared. At the present, the concept of “cluster” includes all chemical compounds that are intermediate between a molecule and a bulk solid with the most varied stoichiometric composition and geometric structure. In modern chemistry, clusters also include nanoparticles of an ordered structure with a given packing of atoms and a regular geometric shape. Moreover, the concept of “cluster” has now gone beyond the framework of chemical science and has found its application in completely different fields of science and technology, which are practically not connected with chemistry. Currently, an “extended” interpretation of this term meaning the combination of several homogeneous elements, which can be considered as an independent unit with certain properties, has become widespread. Examples of such a “synthetic” approach are the word combinations “star cluster” in astronomy, “computer cluster” in computer science, “tone cluster” in music, “cumulonimbus cluster” in meteorology, etc. In addition, although the author of these lines and concurrently Guest Editor of the Special Issue indicated above (see photo right) does not feel any joy about such an “extended” interpretation of the term “cluster,” it will hardly be possible to exclude it from general use. That is why, in the future, for greater certainty and accuracy, instead of the word “cluster,” the concept “metal cluster” will be used, which means any chemical substance where there is precisely a chemical metal–metal bond, regardless of what the nature of these metals is, whether they are the same or different, whether they are connected or not by chemical bonds with non-metals, etc. In addition, be that as it may, chemical compounds that fall under the abovementioned classical definition of “cluster” (i.e., containing at least one metal-metal chemical bond) are still of very significant interest, and this interest increases from year to year.

The abovementioned definition of “cluster” was given by Cotton in 1964, however, the first metal cluster compound that became known to man and found the practical application was probably dimercury dichloride (“calomel”) Hg_2_Cl_2_, which was known in India already since the 12th century; despite this, the existence of a metal-metal bond (namely, Hg–Hg) in it, as well as in a number of other metal chlorides and metal chloride complexes (e.g., in [M_2_Cl_9_]^3-^, where M = Mo, W, and Re), was established only in the twentieth century thanks to the use of X-ray diffraction analysis. By the way, thanks to the use of, namely, this physicochemical method, it became clear that a number of “simple” chemical compounds, upon closer consideration, are clusters; classic examples here are chromium(II) and copper(II) acetates with the simplest formula M(CH_3_COO)_2_, each of which is actually a dimeric compound with a chromium–chromium and copper–copper bond, respectively, and, therefore, their real composition should be represented by the formula M_2_(CH_3_COO)_4_ (M = Cr, Cu), and palladium dichloride with the simplest formula PdCl_2_, which is, in fact, a hexanuclear compound Pd_6_Cl_12_ with an octahedral carcass Pd_6_^12+^ and Pd–Pd bonds between the palladium atoms contained in its composition.

Despite the above limitation, the number of objects falling under the concept of “metal cluster” remains very significant. To a first approximation, the following two categories of such objects can be distinguished–metal clusters in which a carcass of metal atoms is bonded to certain organic and/or inorganic compounds (so-called ligands) and metal clusters in which such binding is absent (they can be called “ligand clusters” and “ligand-less clusters,” respectively). Metal clusters of the second category, in turn, can also be subdivided into two groups, i.e., purely metallic, containing only atoms of metal elements, and nonmetal-metallic, containing, along with atoms of metal elements, atoms of nonmetal elements (sulfur, carbon, phosphorus, etc.). In the last 25 years, in connection with the development of nanotechnology, the term “nanocluster” (which is actually a synonym for a cluster in the broad sense of the word) has come into wide use, as a result of which such objects as molecular clusters, colloidal clusters, and solid-state nanoclusters (moreover, not only based on metals but also on nonmetallic components), were combined into a unified totality. Taking into account the above restrictions, molecular nanoclusters should be considered as either polynuclear coordination compounds, which are a skeleton of metal atoms surrounded by ligands, or ligandless metal clusters; colloidal nanoclusters, as nanoparticles of elemental metals formed as a result of chemical reactions in solutions; and solid-state nanoclusters, as nanoparticles of elemental metals formed as a result of various transformations in the solid phase. A special case of the latter variety of nanoclusters is so-called matrix nanoclusters, which are metal clusters isolated from each other, enclosed in a solid-phase matrix, due to which the processes of their aggregation are prevented.

An important point in the physics-chemistry of clusters, in general, and metal clusters, in particular, is also their subdivision according to the number of metal atoms forming the skeleton of the metal cluster–the so-called nuclearity. According to this indicator, metal clusters are conditionally subdivided into small (with the number of atoms from 3 to 12), middle (with the number of atoms from 13 to 40), large (the number of atoms in which varies from 41 to 100), and super-large or “giant” (the number of atoms in which it exceeds 100). It should be noticed in this connection that the maximal number of metal atoms in a metal cluster, which has been reliably recorded experimentally so far, was over 2000 and took place in the compound of Pd(II) with 1,10-phenanthroline (*phen*) of the formula [Pd_2057_(*phen*)_84_](CH_3_COO)_1600_. However, such a number of atoms in a metal cluster molecule, most likely, is not the limit. Although, in principle, metal clusters with any number of atoms in a structural unit are possible, nevertheless, increased stability of cluster systems, the number of atoms in which corresponded to certain specific numbers, namely, 13, 55, 147, 309, 561, 923 (which even received the name “structural magic numbers”), was long noticed. The modern theory of the structure of metal clusters was able to explain their origin, and one of these numbers, namely 2057, coincides with the number of metal atoms in the largest known metal cluster. In principle, other variants of the systematics of metal clusters are also possible, e.g., according to the assortment of metal atoms forming them (homonuclear and heteronuclear), according to the total charge of their molecules (cationic, neutral, and anionic), according to the specifics of the coordination polymers from which their carcasses are composed, etc.

It is possible to talk about the features of this very peculiar category of chemical objects for a very long time; however, even the above is quite enough in order to unequivocally assert that already from a purely academic point of view, they deserve the closest attention from both chemists and representatives of branches of science related to chemistry. Their significance becomes even higher if we take into account the fact that, at present, they found many applications in various areas of anthropogenic activity, and their scope is expanding from year to year. First of all, this concerns industrial organic synthesis, e.g., hydrogenation and oxidation reactions, in which these compounds combine the advantages of homogeneous and heterogeneous catalysts. There are known examples of catalysis by metal clusters of some natural processes, i.e., so, molecular nitrogen is transformed into ammonia on the Fe–Mo–S metal cluster, which is one of the fragments of the nitrogenase enzyme; carbon monoxide is oxidized to carbon dioxide with the participation of enzymes containing Fe_2_ or FeNi metal clusters; and ferredoxins containing Fe_2_S_2_, Fe_3_S_4_, and Fe_4_S_4_ metal clusters play an important role in the transfer of electrons in some metabolic processes. Some metal-clusters, in particular PbMo_6_S_8_, are superconducting materials with high critical magnetic fields. Metal clusters are components of a number of so-called metal-polymers, which, along with high thermal stability and mechanical characteristics, also have unusual magnetic and electrical properties.

With increasing attention to these chemical compounds, it is becoming an increasingly important task to predict the physicochemical characteristics of metal clusters that determine their abovementioned useful properties, and above all, to determine the geometric parameters of their molecular structures. Despite the fact that a unified theory of chemical bonding in metal clusters is currently under development, the above problem is already being successfully solved due to the availability of modern quantum chemical calculation methods, as well as computer technologies and related experimental equipment. Among the most promising methods for calculating molecular structures and associated physicochemical parameters of metal clusters is the method based on the ideas of density functional theory (DFT), which is now becoming increasingly popular among chemists. Nevertheless, there are still not enough theoretical works devoted to the quantum-chemical calculations of the above metal complexes by the DFT method, as well as more advanced calculation methods, in the literature–there are much less of them than works devoted only to the synthesis of these compounds. Part of the reason for this is connected with the complexity of the molecular structures of metal clusters, as well as in the pronounced ability to manifest a wide variety of isomerism types, as a result of which the quantum-chemical calculation of these compounds using even the DFT method with the simplest basis sets, not to mention more advanced theoretical methods (in particular, CCSD (T) and QCISD (T)), in most cases, is very time consuming and therefore very difficult for practical implementation. However, there is no doubt that with the improvement of computer technology, this method will become more and more accessible for use in scientific work, including in relation to metal clusters. The present Special Issue of ***Molecules***, as it seems to the author of these lines, is intended to, at least to some extent, fill this still-existing gap in theoretical and quantum chemistry in this specific kind of chemical compounds. In addition, the author of this Editorial would like to hope very much that the idea of creating this special issue will find wide support from its readers and will contribute to the emergence of new ideas and materials, valuable comments, and suggestions in this—undoubtedly promising!—scientific direction.

## Data Availability

No new data were created or analyzed in this study. Data sharing is not applicable to this article.

